# Dynamics of rumen bacterial composition of yak (*Bos grunniens*) in response to dietary supplements during the cold season

**DOI:** 10.7717/peerj.11520

**Published:** 2021-06-18

**Authors:** Anum Ali Ahmad, Jian Bo Zhang, Zeyi Liang, Chao Yang, Qudratullah Kalwar, Tariq Shah, Mei Du, Ishaq Muhammad, Juanshan Zheng, Ping Yan, Xue-Zhi Ding, Ruijun Long

**Affiliations:** 1 State Key Laboratory of Grassland Agro-Ecosystems, School of Life Sciences, Lanzhou University, Lanzhou, China; 2 Key Laboratory of yak Breeding Engineering, Lanzhou Institute of Husbandry and Pharmaceutical Sciences, Chinese Academy of Agricultural Sciences, Lanzhou, China; 3 CAS Key Laboratory for Agro-Ecological Processes in Subtropical Region, National Engineering Laboratory for Pollution Control and Waste Utilization in Livestock and Poultry Production, Hunan, Provincial Engineering Research Center for Healthy Livestock and Poultry Production, South-Central Experimental Station of Animal Nutrition and Feed Science in Ministry of Agriculture, Institute of Subtropical Agriculture, Chinese Academy of Sciences, Changsha, Hunan, China; 4 Key Laboratory of Veterinary Pharmaceutical Development, Ministry of Agricultural and Rural Affairs, Lanzhou Institute of Husbandry and Pharmaceutical Sciences, Chinese Academy of Agricultural Sciences, Lanzhou, China

**Keywords:** Yak, Rumen microbiota, Grazing, Dietary supplement, Rumen fermentation

## Abstract

This study aimed to explore the rumen bacterial community of yak in response to dietary supplements during the cold season. In addition, the rumen fermentation products were also analyzed. Twenty-one female domestic yaks were randomly divided into three groups i.e., pure grazing (GG) group, grazing plus oats hay supplement (OG) group, and grazing plus concentrate supplement group (CG). Rumen contents were collected after 90 days to assess rumen fermentation parameters and bacterial community. The GC group presented higher concentrations of ammonia nitrogen (*P* < 0.001), and total volatile fatty acids (TVFA) (*P* < 0.001), and lower rumen pH (*P* < 0.001) compared to other experimental groups. The CG group displayed higher proportions of propionate, butyrate, isobutyrate, and isovalerate while lower A/P ratio compared to other experimental groups. Shannon, Chao1, and ACE values were significantly lower in the OG group compared to GG and CG groups. Anosim test showed significant differences in bacterial community structure between groups but the PCA plot was not very informative to see these differences. Bacteroidetes, Proteobacteria, and Firmicutes were the three dominant phyla in all groups. The genera *Oscillospira* was more abundant in GG and OG groups. Higher relative abundance of *Ruminococcus* and *Clostridium* was observed in the GG group, while *Ruminobacter, Corynebacterium,* and *Selenomonas* were more abundant in the CG group. These findings will help in improving our understanding of rumen bacteria in yaks in response to changes in diet.

## Introduction

The rumen is an anaerobic tank containing different types of microbes to help ruminants in the digestion of feed. Rumen microbiota converts solid fiber into volatile fatty acids, which are an important energy source for the ruminants (Yohe et al., 2019). Thus, rumen microbiota plays a key role in the maintenance, immunity, and growth performance of ruminants (Malmuthuge, 2017; Schären et al., 2018). Studies have reported that many factors can contribute to define the rumen microbiota, specifically diet (Tremaroli & Bäckhed, 2012; Wang et al., 2018). Several studies have reported distinct variations in the rumen microbial community of cows when fed different dietary fiber (Thoetkiattikul et al., 2013) and diets with different concentrate to forage ratios (Wang et al., 2020), indicating a clear influence of diet on the microbiota. So, a comprehensive understanding of the rumen microbial community and what are the changes it undergoes due to different type of diets is important to enhance the welfare, health, and production of livestock.

Yak (*Bos grunniens*), a noteworthy ruminant species of Qinghai Tibetan Plateau, plays a vital part in the daily life of local herdsmen by providing them milk, meat, fuel, and economic benefits (Dong et al., 2006). Yaks mainly rely on natural forage of local alpine pastures to meet their nutrient requirements and suffer from bodyweight variations due to seasonal fluctuations in forage supply (Long et al., 2008). During the cold season, inadequate forage and low temperature result in reduced nourishment, slow growth, weight loss, and even death of yaks as well as an economic loss (Xue, Zhao & Zhang, 2005). Yaks are provided with supplements during the cold season to improve their growth performance and productivity. Supplements provide energy, minerals, fatty acids, vitamins, and proteins to yak and reduce their weight loss (Grilli et al., 2016). Among other supplements, oats hay, and concentrate have been extensively used by herdsmen during the cold season in recent years.

Several studies have studied rumen microbiota of yak (Guo et al., 2015; Guo et al., 2020; Ren et al., 2020) and alterations in rumen microbiota of yak in response to variations in the diet have been an area of focus (Ahmad et al., 2020; Yang et al., 2020). For example, the effect of influence of the concentrate to forage ratio on rumen microbiota of yak has been investigated (Liu et al., 2019a). Similarly, the influence of total mixed ration on bacterial diversity of yak was compared with pure grazing yak during the warm season (Fang et al., 2015). However, less knowledge is available on the effect of dietary supplements on the rumen bacterial community of grazing yak during the cold season. Therefore, the objective of the current study was to evaluate the bacterial rumen community of yak in response to dietary supplements during the cold season. Moreover, we also analyzed the rumen fermentation products. We hypothesized that dietary supplements will alter the bacterial composition of yak and in turn, rumen fermentation products will also be altered. This study will benefit in improving our understanding of variations in rumen bacteria in yaks in response to changes in diet.

## Material and Methods

### Ethics statement and site description

All procedures involved in animal care and their use were in strict agreement with the guidelines approved by the Animal Administration and Ethics Committee of Lanzhou Institute of Husbandry and Pharmaceutical Sciences (LIHPS) of CAAS, China (SYXK-2018-0011). After collection, the samples were processed strictly in agreement with the rules of LIHPS. The yaks were delivered for experiments by the yak owner and after the completion of the experiment, all yaks keep on living for other experimental work.

The feeding trial was conducted for 90 days from December 2018 to February 2019 at Hezuo Yak Breeding Cooperatives (35°08′N, 102°99′E, 2,960 m above sea level) of the Tibetan Autonomous Prefecture of Gannan, Gansu province, China. The pastures in the area was characterized by alpine meadows and inhabited by vegetation species of *Kobresia graminifolia*, *Kobresia humilis*, *Elymus nutan*, *Kobresia pygmaea*, *Anaphalis lacteal*, *Polyginum viviparum*, *Potentilla fruticose*, *Cortaderia jubata,* and *Sibiraea angustata*. The average temperature and precipitation during trial were −3.9 °C and 4.8 mm, respectively.

### Animal management, diets, and experimental design

Twenty-one 3-year old female Gannan yaks having an initial average body weight of 178 ± 2.71 kg from the same herd were randomly placed into three dietary treatment groups (*n* = 7). The animals were lodged individually in tie-stalls and had 9 m^2^ space with bedding in the night for regular activities. The experiment started after 15 days of adaptation period to familiarize the animals with diet, facilities, and staff. All animals remained together in the same flock and three groups grazed freely in the natural alpine pastures from 7:00 to 18:00 with free access to water. To check pasture availability, the height of dominant species from pasture was randomly measured with a ruler. About 10 samples were collected by hand as representative of forage selected by yak, weighted, and dried to calculate dry matter.

The first group served as a control group and grazed the natural pasture without any supplement (GG). The grazing yaks in the second group were supplemented with 1 kg/d oats hay (OG), while grazing yaks in the third group were fed with 0.9 kg/d concentrate pellets (CG), which was based on the amount of feed voluntarily consumed by yaks during adaptation period to ensure the complete absence of orts. The concentrate supplement consisted of corn 500 g/kg, wheat bran 220 g/kg, rapeseed cake 240 g/kg, urea 16 g/kg, salt 17 g/kg, additives 1.5 g/kg, calcium hydrogen phosphate 5.5 g/kg, calcium 5.6 g/kg and phosphorous 2.6 g/kg on DM basis. The nutritional composition of pasture herbage, oats hay, and concentrate pellets is listed in Table 1. Before this study, all yaks only grazed natural pasture and did not receive any supplement and during the experimental period, about 2–3 yaks grazed per hectare of pasture. The yaks were offered oats hay and concentrate pellets twice a day at 07:00 and 18:00**.**

**Table 1:** Nutrient composition of pasture herbage and supplements used during the experiment.

Nutrient composition (g/kg DM)	Pasture herbage	Oats hay	Concentrate
CP	48.2	83.4	118.4
NDF	678.2	595.2	456.1
ADF	423.5	367.3	250.4
DE (MJ/kg DM)	5.25	10.4	18.9

**Notes.**

DE was calculated according to Tables of Feed Composition and Nutritive Values in China (Chinese Feed Database, 2010).

CP crude protein NDF neutral detergent fiber ADF acid detergent fiber DE digestible energy

Forage DMI was measured with lignin as an internal marker and chromic oxide as an external marker as described previously (Sampaio et al., 2011). For this purpose, forage and fecal samples were collected (Ding et al., 2010). Each yak was given 20 g of chromic oxide in a gelatin capsule orally once daily for 9-days preliminary dosing period and 10-days fecal sampling period. The fecal samples were collected daily during the period of administration of chromic oxide. The samples were dried at 60 °C for 48 h, ground to pass through 1 mm sieve, and concentrations of internal and external markers were measured (Myer et al., 2015; Van Soest, Robertson & Lewis, 1991). Forage DMI was estimated from the fecal output of the internal marker adjusted for the supplement contribution. Finally, the total dry matter intake of supplemented groups was calculated by adding forage and supplement intake (Kartchner, 1980).

### Sample collection

The mixed forage samples were taken from the pasture grazed by yaks. The quadrat of 0.5 × 0.5 m^2^ size was randomly placed across the pasture to mimic the yak selection of forage to graze. The forage samples were clipped in triplicates and stored for later nutritional composition analysis.

Rumen sample (liquid contents, 100 mL/yak) was taken at the end of the feeding trial via an oral stomach tube in the morning before feeding to explore rumen fermentation and bacterial composition. The tube was properly washed with fresh water every time before taking a new sample and the first 15–20 ml of the sample was always discarded to prevent saliva contamination. The rumen contents were immediately stored in liquid nitrogen and then preserved at −80 °C for future analysis.

### Nutrient analysis

Pasture forage and feed samples were dried at 65 ^∘^C for 72 h in an oven, pulverized, and passed through a 1 mm sieve. Dry matter (DM) was measured by drying the samples at 135 °C for 3 h (AOAC # 930.15). Crude protein (CP, 984.13) was measured by AOAC methods (Association of Official Analytical, 1990). The neutral detergent fiber (NDF) and acid detergent fiber (ADF) were measured according to the method reported by Van Soest (Van Soest, Robertson & Lewis, 1991). The sodium sulfite was used in the NDF method and the values were corrected for ash content.

### Rumen fermentation parameters analysis

Rumen pH was measured immediately after sampling with a portable pH meter (Kadiya, 6010, Shenzhen, China). The ammonia nitrogen (NH_3_-N) concentration was determined by a colorimetric method (Weatherburn, 1967). The VFA concentration was determined as previously described in (Ahmad et al., 2020). Briefly, the frozen rumen liquid sample was thawed, mixed by vortexing, and five mL of rumen liquid was centrifuged at 3000 × g for 10 min. In a centrifuge tube, 1 mL of supernatant was taken and 0.2 mL of a metaphosphoric acid solution was added to it. The solution was mixed and held for 30 min in an ice bucket and then centrifuged at 1,0000× g at 4 °C. The supernatant was taken into a new centrifuge tube and preserved for testing at 4 °C. The gas chromatography (7890A GC system Agilent Technologies Inc, Santa Clara, CA, USA) with FID detector was used to measure VFA concentration. The gas chromatographic conditions and subsequent test procedures were conducted as described previously (Isac et al., 1994).

### DNA extraction and PCR amplification

The metagenomic DNA of 21 rumen fluid samples was extracted by the cetyltrimethylammonium bromide method. DNA was dissolved in the 200 µL of elution buffer and then preserved at −20 °C. DNA integrity and concentration were determined by 1.5% agarose gel electrophoresis and NanoPhotometer^®^ spectrophotometer (Implen, Westlake Village, CA, USA), respectively. For rumen bacterial analysis, universal primer pairs (515F-806R) with barcodes were used to PCR amplify the V4 region of the 16S rRNA gene (Li et al., 2016). PCR amplification was done by using Phusion^®^ High-Fidelity PCR Master Mix with GC Buffer from New England BioLabs. The amplified products were detected by 2% agarose gel electrophoresis and purified by QIAquick PCR Purification Kit to build libraries. The libraries were built according to the manufacturer’s protocol using TruSeq^®^ DNA PCR-Free sample preparation kit. The libraries were then quantified by using Qubit dsDNA High Sensitivity Assay kit by Invitrogen. The Illumina HiSeq2500 PE250 system was used for paired-end sequencing using the standard protocol.

### Sequencing and data processing

Raw sequences were prepared and QIIME (Quantitative Insights Into Microbial Ecology, Version 1.7.0) software was used for their analysis (Caporaso et al., 2010). After sequencing, primer sequences, barcodes, and low-quality sequences were truncated (Li et al., 2016). Chimeric sequences were identified and discarded from the dataset using UCHIME Algorithm with reference to Gold database to finally get effective tags (Edgar et al., 2011). UCLUST software version 7.1 (http://drive5.com/uparsel/) was used to cluster effective tags into OTUs with 97% identity (Edgar, 2013) and bacterial taxa were identified from Greengenes database using representative sequences (Wang et al., 2007).

Alpha diversity indices such as Chao1, ACE, Simpson, Shannon, and Goods-coverage were calculated using QIIME software. STAMP (Statistical analysis of taxonomic and functional profiles, Version 2.1.3) was used to construct a principal component analysis (PCA) plot to illustrate the significant difference between samples at the OTU level. A correlation heat map was generated in GraphPad Prism version 8.00 for Windows (http://www.graphpad.com/).

### Statistical analysis

Intake data, rumen fermentation parameters and relative abundances of bacterial taxa (Phylum and genus levels) were analyzed using the mixed procedure in SPSS software (Version 20.0) (IBM, Armonk, NY, United States). The dietary group was considered as a fixed effect and the individual yak was taken as a random effect. Residuals were checked for normality using Shapiro–Wilk test. When the residuals did not follow a normal distribution the data was log-transformed. Bonferroni correction method was used for multiple comparisons of groups (Haynes, 2013). Analysis of similarities (Anosim) was performed in R studio by using anosim function of the vegan package to examine the grouping variations of each group separately in the PCA plot. Spearman’s correlation coefficient of rumen fermentation parameters with the 10 more abundant ruminal bacterial genera and genera significantly affected by the treatments were calculated using GraphPad Prism 8.0.2 and a heat map was also generated. Significance was declared at P <0.05 and *P* values were modified using a false discovery rate to exclude false-positive results.

**Table 2:** Total dry matter intake and rumen fermentation parameters of yak supplemented with oat hays and concentrate during the cold season.

Items	Treatments			SEM	*P*-value
	GG	OG	CG		
TDMI (kg/d)	3.32^a^	3.57^b^	4.51^c^	0.124	<0.001
Rumen pH	7.02^a^	6.91^b^	6.76^c^	0.026	<0.001
NH3-N (mg/mL)	5.86^a^	8.61^b^	10.9^c^	0.486	<0.001
TVFA (mM)	52.43^a^	59.41^b^	63.75^c^	1.108	<0.001
Molar proportion of VFA (%)					
Acetate	78.24^a^	76.71^ab^	75.24^b^	0.578	0.031
Propionate	15.13^a^	15.87^a^	17.23^b^	0.302	0.001
Butyrate	4.84^a^	5.56^a^	6.74^b^	0.254	0.001
Isobutyrate	0.85^a^	0.88^a^	0.95^b^	0.012	<0.001
Isovalerate	0.90^a^	0.93^a^	1.25^b^	0.039	<0.001
A/P	5.18^a^	4.84^a^	4.3^b^	0.103	<0.001

**Notes.**

Values are presented as mean ± SEM. Means in a row with different small letter superscripts differ significantly (*P* > 0.05), same letter superscripts present no difference (*P* < 0.05).

GG grazing group OG oats hay supplement group CG concentrate supplement group TDMI total dry matter intake NH3-N ammonia nitrogen TVFA total volaile fatty acids A/P acetate/propionate ratio VFA volatile fatty acids SEM standard error of the mean

**Figure 1:**
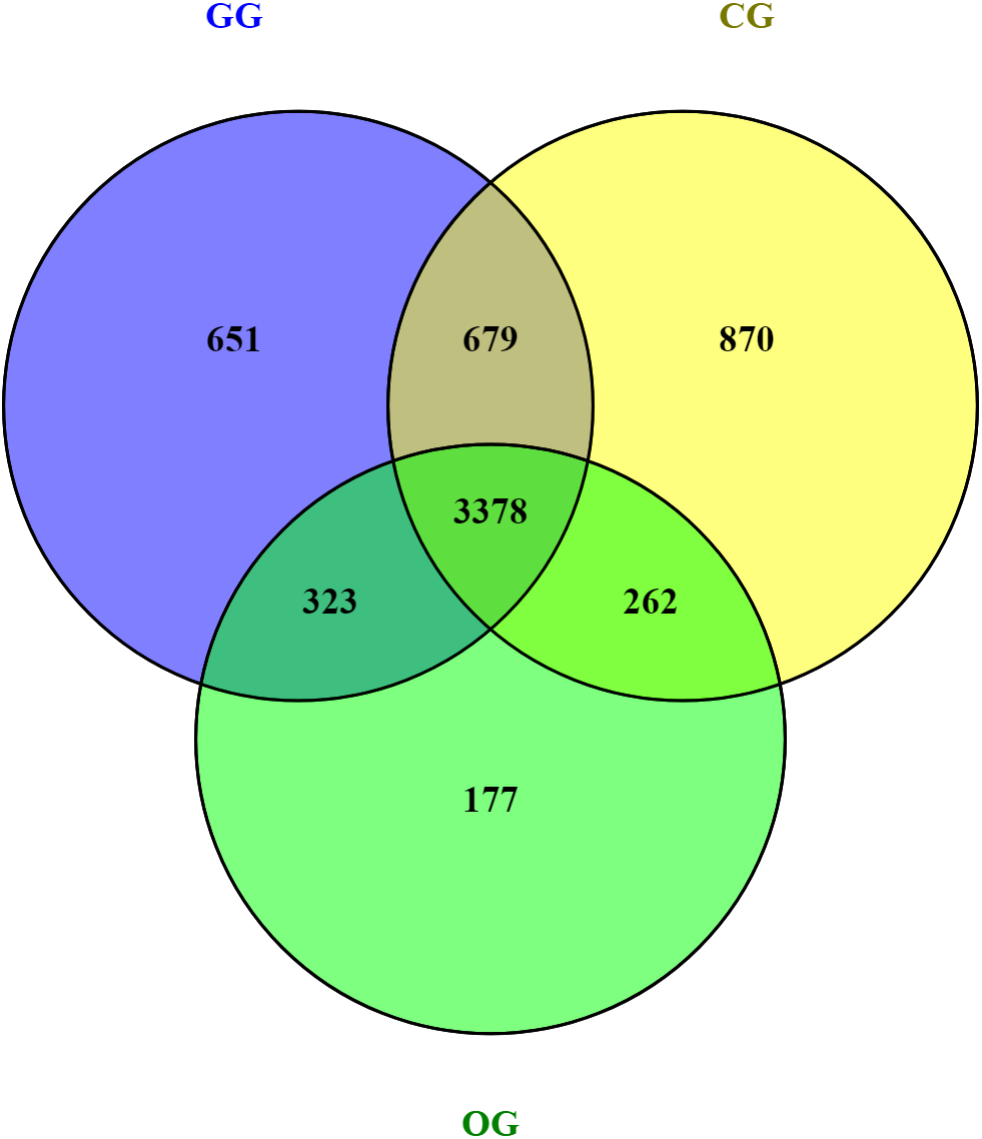
Venn diagram showing operational taxonomic units shared between the three experimental groups (full grazing group (GG); group supplemented with oats hay (OG) and group supplemented with concentrate (CG)). GG, grazing group; OG, oats hay supplement group; CG, concentrate supplement group.

### Data availability

The sequencing data for this study was stored in the European Nucleotide Archive (ENA) at EMBL-EBI under accession number PRJEB36248.

## Results

### Rumen fermentation parameters

Total dry matter intake (TDMI) of the CG group was significantly higher compared to GG and OG groups (Table 2). The results of rumen fermentation parameters are displayed in Table 2. In comparison to GG and OG groups, the CG group showed significantly (*P* < 0.01) higher concentrations of NH_3_-N and TVFA in rumen fluid. OG group also showed higher concentrations of NH3-N and TVFA in rumen fluid than GG group. GG group showed a higher concentration of acetate (*P* = 0.03) than CG groups, while both groups displayed no significant differences with the OG group. GG and OG groups showed significantly lower concentrations of propionate (*P* = 0.01), butyrate (*P* = 0.01), isobutyrate (*P* < 0.01), and isovalerate (*P* < 0.01), and AP ratio (*P* < 0.01) than CG group. However, no significant difference was observed between GG and OG groups. Rumen pH was significantly (*P* < 0.001) lower in the CG group followed by OG and GG groups.

### Bacterial diversity analysis

After filtering, quality control, and chimera removal, a total of 1,097,941 high-quality bacterial sequences were produced and the Good’s coverage was more than 95.2% with an average sequence read length of 253 bp. The rarefaction curve reached the plateau, indicating that the OTUs number did not increase with the increasing size of data and the sequencing data volume was sufficient. The clean reads were clustered into 6,340 OTUs on a 97% sequence similarity basis. The analysis of OTU recognized 3,378 common OTUs in three groups, while 651, 177, and 870 unique OTUs were identified in the GG, OG, and CG groups, respectively (Fig. 1).

Simpson index did not show a significant difference among groups (*P* = 0.21, Table 3). Shannon, chao1, and ACE indices were significantly lower in the OG group compared to GG and CG groups, while no significant variations were observed between GG and CG groups.

**Figure 2:**
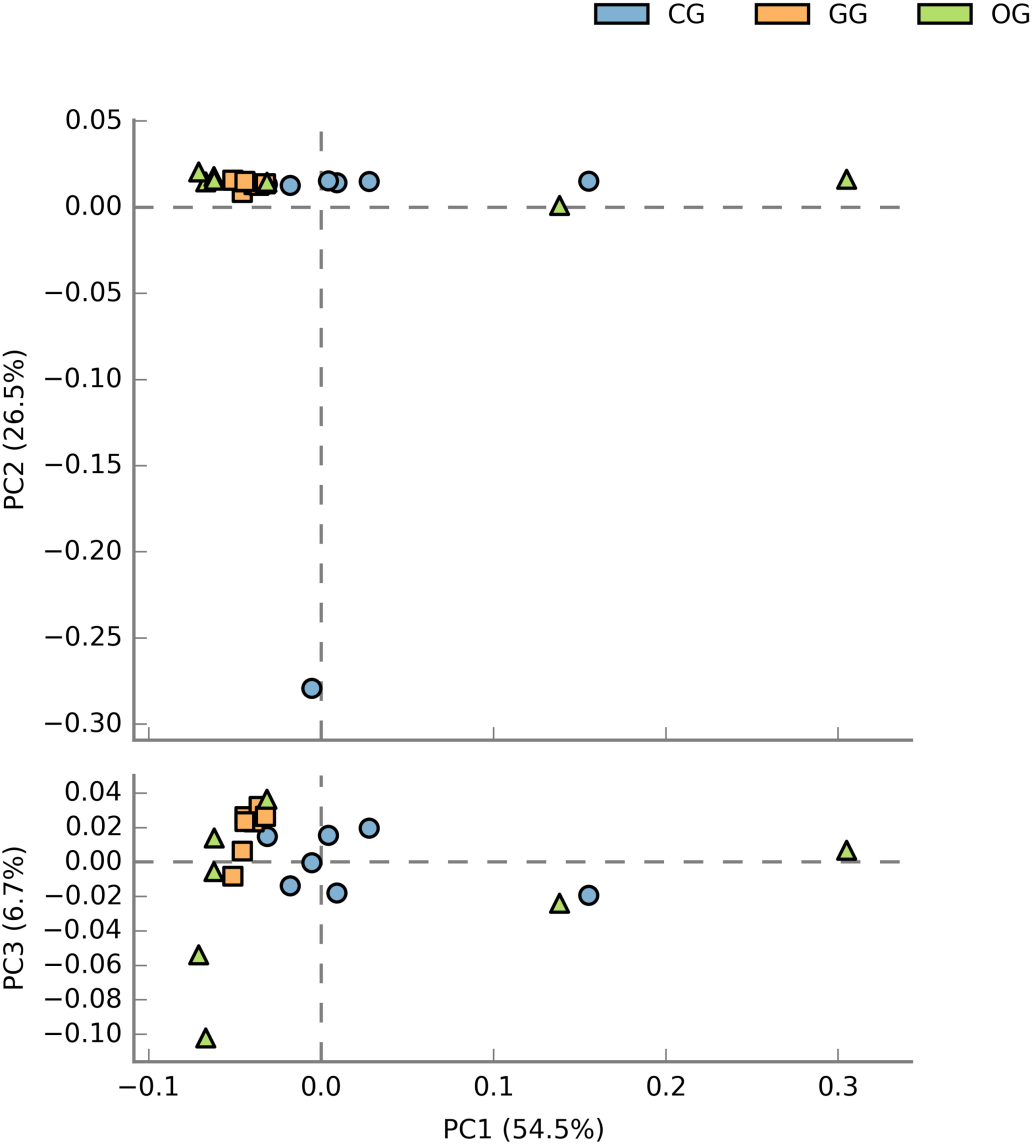
Principal component analysis (PCA) of bacterial community at OTU level of yak supplemented with oats hay and concentrate during cold season. PC1, 1st principal component PC1, 2nd principal component and PC3, 3rd principal component. The percentage of variation explained by each principal coordinate is indicated on the axes. A dot represents each sample and different colors represent different groups. GG, grazing group; OG, oats hay supplement group; CG, concentrate supplement group.

The STAMP software was used to further analyze the difference in the bacterial community among groups by the PCA plot (Fig. 2). PCA plot was not very informative in studying the differences among groups as all the samples assembled close to each other. However, Anosim analysis displayed a significant difference in the composition of rumen bacteria at OTU level between GG and CG (*R* = 0.44, *P* < 0.001) groups, between GG and OG (*R* = 0.21, *P* = 0.005) groups and between CG and OG (*R* = 0.18, *P* = 0.038) groups.

**Table 3:** The alpha diversity indices of yak supplemented with oat hays and concentrate during the cold season.

Item	Treatments			SEM	*P*-value
	GG	OG	CG		
Shannon	9.22^a^	7.96^b^	8.34^a^	0.183	0.008
Simpson	0.99	0.96	0.97	0.0072	0.21
chao1	2682.89^a^	2067.27^b^	2658.97^a^	89.844	0.002
ACE	2779.28^a^	2142.96^b^	2767.94^a^	93.522	0.002

**Notes.**

Values are presented as mean ± SEM. Means in a row with different small letter superscripts differ significantly (*P* < 0.05), same letter superscripts present no difference (*P* > 0.05).

GG grazing group OG oats hay supplement group CG concentrate supplement group

### Rumen bacterial composition

The taxonomic analysis showed that the yak rumen bacterial community comprised of 23 phyla and 487 genera. At the phylum level, Bacteroidetes (42.0% ± 9.1%), Proteobacteria (14.6% ± 11.0%) and Firmicutes (15.9% ± 3.34%) were found to be the most dominant phyla in three groups (Fig. S1). Verrucomicrobia (6.9% ± 2.7%), Fibrobacteres (4.8% ± 2.7%), and Tenericutes (4.2% ± 5.2%) were other abundant phyla present in three groups. The low abundance phyla among three groups included Cyanobacteria, Actinobacteria, Acidobacteria, and Lentisphaerae.

Changes at the phylum level in the three groups are shown in Table 4. The abundance of Tenericutes in the OG group was significantly lower (*P* = 0.02) compared to CG and GG groups, while no significant variations were recorded between GG and CG groups. The phylum Cyanobacteria was significantly higher (*P* = 0.04) in the CG group compared to the OG group, while the GG group showed no significant difference with OG and CG groups.

**Table 4:** Relative abundance of bacteria at the phylum level in yak rumen supplemented with oat hays and concentrate during the cold season.

Taxonomy	Log transformed values			SEM	*P*-value
	GG	OG	CG		
Bacteroidetes	1.64	1.61	1.57	0.022	0.408
Firmicutes	0.93	1.02	1.22	0.065	0.169
Proteobacteria	1.25	1.14	1.17	0.207	0.063
Verrucomicrobia	0.84	0.83	0.77	0.036	0.733
Fibrobacteres	0.59	0.61	0.65	0.054	0.914
Tenericutes	0.72^a^	0.52^b^	0.55^a^	0.034	0.023
Cyanobacteria	0.086^ab^	−0.004^a^	0.26^b^	0.046	0.048
Actinobacteria	−0.081	−0.34	−0.029	0.0649	0.094
Acidobacteria	−0.27	−0.74	−0.53	0.095	0.144
Lentisphaerae	−0.069	−0.009	−0.048	0.0220	0.552

**Notes.**

Values are presented as mean ± SEM. Means in a row with different small letter superscripts differ significantly (*P* < 0.05), same letter superscripts present no difference (*P* > 0.05).

GG grazing group OG oats hay supplement group CG concentrate supplement group

To further explore the microbial abundance, classification was performed at the genus level. *Prevotella* (16.2% ± 6.4%) was the dominant genus followed by *Fibrobacter* (4.84% ± 2.7%), *Ruminobacter* (2.18% ± 2.4%) and *CF231* (1.6% ± 0.03%), accounting for 24.8% of all genera (Fig. S2). Other dominant genera included *RFN20* (1.25% ± 0.04%), *Anaeroplasma* (1.0% ± 0.03%), *BF311* (1.6% ± 0.96%), *Halomonas* (1.6% ± 0.83%), *Bacteroides* (0.29% ± 0.05%) *and Lactobacillus* (0.22% ± 0.07%).

The variations in the relative abundance of the top 20 genera in three groups were examined (Table 5). The abundance of *Ruminobacter* was significantly (*P* < 0.01) lower in GG compared to OG and CG groups. However, no significant difference was displayed between OG and CG groups. The relative abundance of *Oscillospira* was significantly (*P* = 0.014) higher in the GG group compared to the CG group, while no significance was recorded between GG and OG and between OG and CG groups. The relative abundance of *Ruminococcus* (*P* = 0.04) was significantly higher in the GG group compared to the OG group, while no significant difference was observed between GG and CG groups and between OG and CG groups. The abundance of *Clostridium* (*P* = 0.007) was significantly higher in the GG group compared to OG and CG groups. However, no significant difference was observed between OG and CG groups. The genera *Selenomonas* (*P* = 0.014)*,* and *Corynebacterium* (*P* = 0.043) were significantly higher in the CG group compared to GG and OG groups, while no difference was observed between GG and OG groups.

**Table 5:** Relative abundance of the 20 more representative genera in yak rumen supplemented with oat hays and concentrate during the cold season.

Taxonomy	Log transformed values			SEM	*P*-value
	GG	OG	CG		
*Prevotella*	1.21	1.14	1.18	0.038	0.769
*Fibrobacter*	0.59	0.61	0.65	0.544	0.914
*Ruminobacter*	−0.22^a^	0.25^b^	0.36^b^	0.091	0.009
*CF231*	0.23	0.20	0.14	0.024	0.342
*RFN20*	0.14	0.001	0.066	0.0359	0.291
*Anaeroplasma*	0.047	−0.097	−0.12	0.0341	0.226
*BF311*	0.029	−0.090	−0.17	0.0556	0.327
*Halomonas*	−0.16	−0.41	−0.023	0.0826	0.156
*Bacteroides*	−0.97	−1.25	−0.85	0.134	0.497
*Lactobacillus*	−0.153	−1.20	−0.85	0.1369	0.128
*Oscillospira*	−0.49^a^	−0.58^ab^	−0.77^b^	0.042	0.014
*Ruminococcus*	−0.0067^a^	−0.16^b^	−0.11^ab^	0.02721	0.042
*Clostridium*	−0.22^a^	−0.37^b^	−0.39^b^	0.026	0.007
*Butyrivibrio*	−0.26	−0.35	−0.34	0.025	0.347
*Treponema*	−0.25	−0.24	−014	0.025	0.143
*Methylobacterium*	−0.58	−0.87	−0.34	0.095	0.065
*Selenomonas*	−0.53^a^	−0.58^a^	−0.28^b^	0.041	0.014
*Corynebacterium*	−1.54^a^	−1.39^a^	−0.78^b^	0.136	0.043
*Kaistobacter*	−0.45	−0.59	−0.36	0.053	0.233
*Succinivibrio*	−0.77	−0.81	−0.68	0.044	0.529

**Notes.**

Values are presented as mean ± SEM. Means in a row with different small letter superscripts differ significantly (*P* < 0.05), same letter superscripts present no difference (*P* > 0.05).

GG grazing group OG oats hay supplement group CG concentrate supplement group

### Correlation analysis between bacterial genera and rumen fermentation parameters

**Figure 3:**
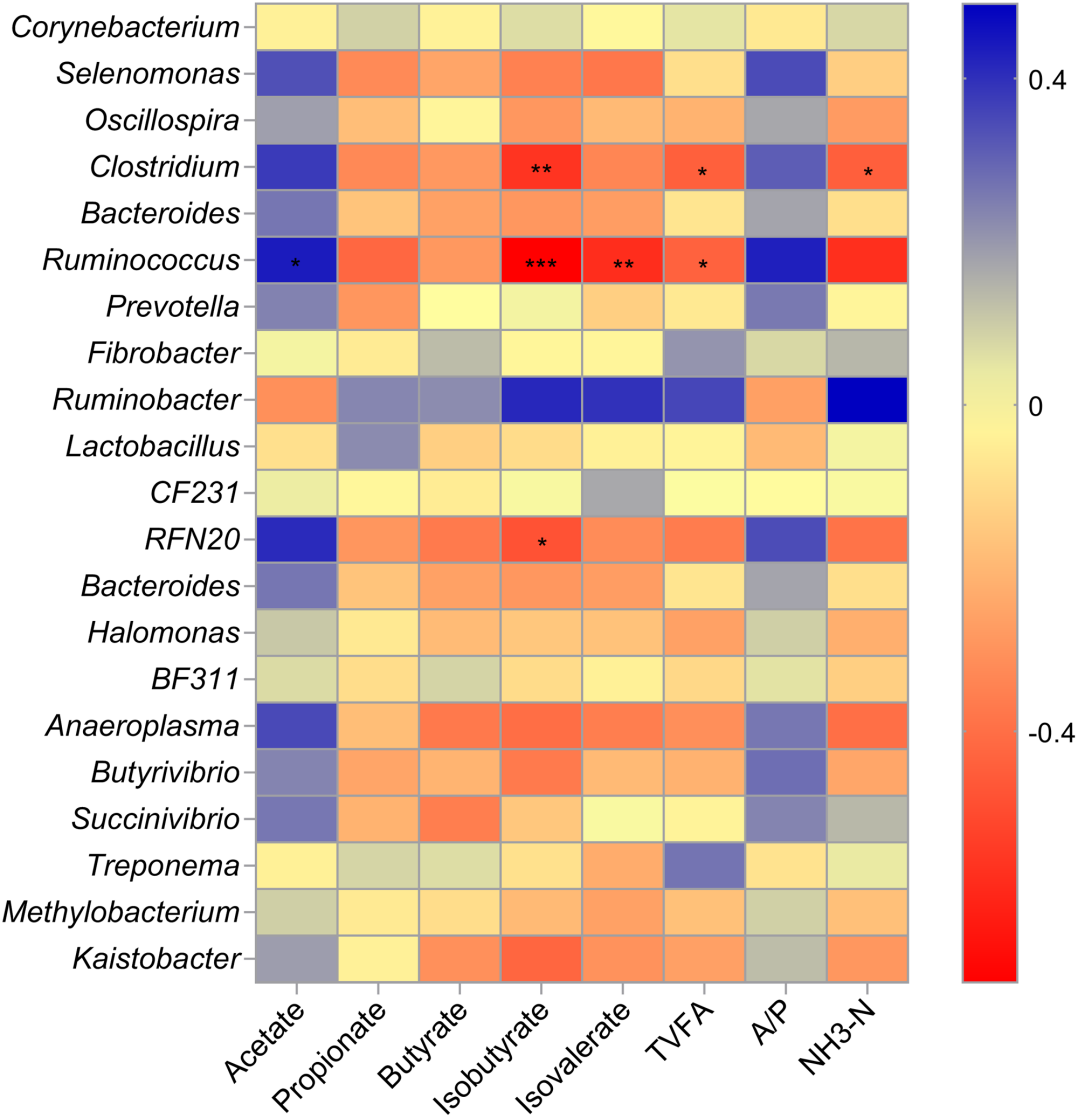
The heat map displaying the correlation of rumen fermentation parameters with relative abundance of bacterial genera. Spearman’s correlation coefficients were calculated and the values between −1 and 1 in the color key indicate negative (red) and positive (blue) correlations. GG, grazing group; OG, oats hay supplement group; CG, concentrate supplement group; TVFA, total volatile fatty acids; A/P, acetate/propionate ratio. * = *P* < 0.05, ** = *P* < 0.01 and *** = *P* < 0.001.

Spearman’s correlation was implemented to identify the association of rumen fermentation parameters with the abundance of top 10 and significantly varied bacterial genera (Fig. 3). Genus *Ruminococcus* was positively correlated with acetate (*r* = 0.44, *P* = 0.04) ruminal proportions and negatively associated with ruminal TVFA (r = −0.43, *P* = 0.016) concentrations and isobutyrate (r = −0.71, *P* < 0.001 0.02), and isovalerate (r = −0.58, *P* < 0.01) proportions. Genus C*lostridium* was negatively correlated with ruminal TVFA (r = −0.44, *P* = 0.04) and NH_3_-N (r = −0.44, *P* = 0.04) concentrations, and isobutyrate (r = −0.56, *P* < 0.01) proportions. Finally, genus *RFN20* was negatively correlated with ruminal isobutyrate (r = −0.48, *P* = 0.02) proportions. The rest of the genera did not show any significant relationship with any parameters.

## Discussion

Volatile fatty acids (VFA) are vital fermentation products of microbes residing in the rumen of ruminants and their generation and proportions depend on diet and rumen microbiota. In this study, we recorded maximum TVFA concentrations and minimum pH in the CG group compared to other groups, which is consistent with other studies when the concentrate was supplemented to grazing yaks (Ash & Dobson, 1963), or when the level of supplemented concentrate increased in the diet (Ahmad et al., 2020). In the CG group, the rapid fermentation of easily available carbohydrates such as starch might increase the concentration of TVFA in the rumen and in turn decreased the rumen pH as compared to other groups. However, rumen pH in the CG group was in a suitable range and did not cause acidosis, which might be because the supplemented concentrate quantity was not very high and the forage to concentrate ratio was not low enough. The shortage of forage availability together with the low quality of grass during the cold season led to lower DMI values in grazing yak (GG), which might result in the observed reduction of TVFA concentrations in the GG group compared to other groups. High forage content in GG and CG groups increased the proportion of acetate and decreased propionate, which is in agreement with other study (Agle et al., 2010). Similar results were reported in the rumen of yak fed high forage diet compared to concentrate diet (Wang et al., 2020) and in a study conducted on Holstein cows when fed a high concentrate diet compared to a high forage diet (Wang et al., 2020). We also recorded higher proportions of isobutyrate and isovalerate in the CG group compared to GG and OG groups, which are similar to results reported in yaks fed concentrate diet (Liu et al., 2019a). Isobutyrate and isovalerate are derived from feed protein degradation and deamination of amino acids (Eugène, Archimède & Sauvant, 2004). In this study, the increase in protein content in the CG group led to an increased concentration of NH_3_-N in the rumen, and could also explained the higher concentrations of these branched-chain fatty acids observed in the CG group. The NH_3_-N is the source of microbial protein and its high concentration in the CG group might be related to the increased production of microbial protein in the rumen (Zhang et al., 2017). Diet not only alters the fermentable substrate but also changes the rumen fermentation patterns and microbial profile (Ghimire et al., 2017), so we assumed that these alterations in rumen fermentation might be related to variations in the rumen bacterial composition of yak.

We then studied the effect of dietary supplements on the rumen bacterial composition of yak. We recorded higher bacterial diversity in GG and CG groups. High diversity values in the CG group are inconsistent with previous reports which showed a decrease in bacterial diversity when fed a concentrate diet (Liu et al., 2019b; Zhang et al., 2017). Low rumen pH is stated to reduce the diversity of rumen bacteria, however, rumen pH in the CG group was in the normal range to cause inhibition of growth of acid-sensitive bacteria (Owens et al., 1998), which might explain the lack of effect of concentrate supplementation on bacterial diversity. We observed significantly reduced bacterial diversity in the OG group compared to other groups, which is inconsistent with previous results reported in yak (Liu et al., 2019a). Rumen microbiota tends to vary with diet, however, core microbiota is found across a wide geographical area (Henderson et al., 2015). We also found a core microbiota in the three diets, being Bacteroidetes, Proteobacteria, and Firmicutes the three dominant phyla in the studied groups, which is consistent with the previous reports on yak (Xue et al., 2016). We did not find any significant variations in the relative abundance of dominant phyla among the three groups. Only two minor phyla Tenericutes and Cyanobacteria were significantly affected by dietary supplements. We observed a high abundance of Tenericutes in the GG group compared to other groups, consistent with studies on sheep and cows (Fu et al., 2020; Thoetkiattikul et al., 2013). However, not much information is available on the role of Tenericutes in the rumen, so a further explanation would be difficult to find. The phylum Cyanobacteria is known for its ability to ferment a variety of sugars into acetate and butyrate (Di Rienzi et al., 2013; Soo et al., 2014). We recorded the high abundance of Cyanobacteria in the CG group, which might be attributed to highest sugar content in the concentrate diet compared to GG and OG groups.

At the genus level, dietary supplements significantly varied the abundance of *Ruminobacter, Oscillospira*, *Clostridium*, *Ruminococcus, Corynebacterium*, and *Selenomonas*. The genus *Ruminobacter* is mainly responsible for degrading starch into acetate and propionate in the rumen (Hamlin & Hungate, 1956). The abundance of this genus in the rumen has been reported to be associated with concentrate diet in Holstein Cows (Wang et al., 2020), which is in agreement with our results. The genus *Oscillospira* is the first reported bacteria to be involved in plant cell wall degradation (Yanagita et al., 2003), which might explain its abundance in GG and OG groups. The higher abundance of this genus in the rumen of cattle, sheep, and goats has been associated with the feeding of forage diets (Hua et al., 2017; Mackie et al., 2003). The genera *Clostridium* and *Ruminococcus* are known as cellulolytic bacterial species that produce a variety of enzymes to degrade cellulose into acetate (Miller & Wolin, 1995; Varel, 1989), which was in agreement with the positive correlation of these genera with acetate in this study. The higher abundance of *Clostridium* and *Ruminococcus* was reported in the rumen of natural grazing yaks (Xue et al., 2016; Yang et al., 2020). Similar results were reported in the rumen of steers fed high fiber feedstuff (Huws et al., 2010), in agreement with their importance in the rumen of the GG group in the present study. The genus *Selenomonas* is known as propionic acid bacteria because it produces propionate by consuming carbohydrates and lactate in the rumen (Stewart, Flint & Bryant, 1997). It is also reported to promote the growth of bacteria and prevent rumen acidosis (Bryant, 1956). Therefore, the high abundance of *Selenomonas* in the CG group might be related to the availability of more fermentable substrate compared to other groups, in agreement with the previous studies (Kim et al., 2018; Hu et al., 2020) who reported that a moderate increase in dietary energy increased the abundance of *Selenomonas* in the rumen of yak. Although in the present study no significant correlation was observed between genus *Corynebacterium* and ruminal butyrate proportions, this genus is known to be able to utilize carbohydrates to produce butyrate in the rumen (Douglas & Gunter, 1946), which might explain the higher proportion of butyrate observed in the CG group compared to other groups. However, the detailed information about its role in the rumen is scarce and it is previously linked with pathogenicity in ruminants (Smith, 1966). Overall, these variations reveal important information linked to the provision of dietary supplements to yak.

## Conclusions

In conclusion, dietary supplementation influenced the rumen microbial composition of yak which in turn influenced the rumen fermentation products. The phyla Bacteroidetes, Proteobacteria and Firmicutes, and the genus *Prevotella* were found to be the core microbiome in all studied groups. Some bacterial taxa were more related to the different diets and also influence the rumen fermentation products. The higher abundance of *Oscillospira*, *Clostridium*, and *Ruminococcus* in the grazing yak and oats hay supplement group can be linked to the higher amounts of fiber in these diets and the consequent higher acetate proportions observed in the rumen of these animals. While, concentrate supplement increased the abundance of *Ruminobacter*, Corynebacterium, and *Selenomonas* which in turn increased the proportions of propionate, butyrate, and branched-chain fatty acids in the rumen of yak.

##  Supplemental Information

10.7717/peerj.11520/supp-1 Supplemental Information 1 The relative abundance of bacteria at phylum level in yak supplemented with oats hay and concentrate *Y*-axis shows the relative abundance of bacteria, while *Y*-axis displayed three different dietary groups.

10.7717/peerj.11520/supp-2 Supplemental Information 2 The relative abundance of bacteria at genus level in yak supplemented with oats hay and concentrate *Y*-axis shows the relative abundance of bacteria, while *Y*-axis displayed three different dietary groups.

10.7717/peerj.11520/supp-3 Supplemental Information 3 The raw data of rumen fermentation parameters of yak supplemented with oats hay and concentrate during cold season GG, grazing group; OG, oats hay supplement group; CG, concentrate supplement group.

## Additional Information and Declarations

### Data Availability

The following information was supplied regarding data availability: The sequencing data for this study are available in the European Nucleotide Archive (ENA) at EMBL-EBI: PRJEB36248.
