# Supplementary figures and images for: Dynamics of rumen bacterial composition of yak (Bos grunniens) in response to dietary supplements during the cold season
Source: PeerJ. 2021 Jun 18;9:e11520. doi: 10.7717/peerj.11520 (PMC8216167; doi:10.7717/peerj.11520)

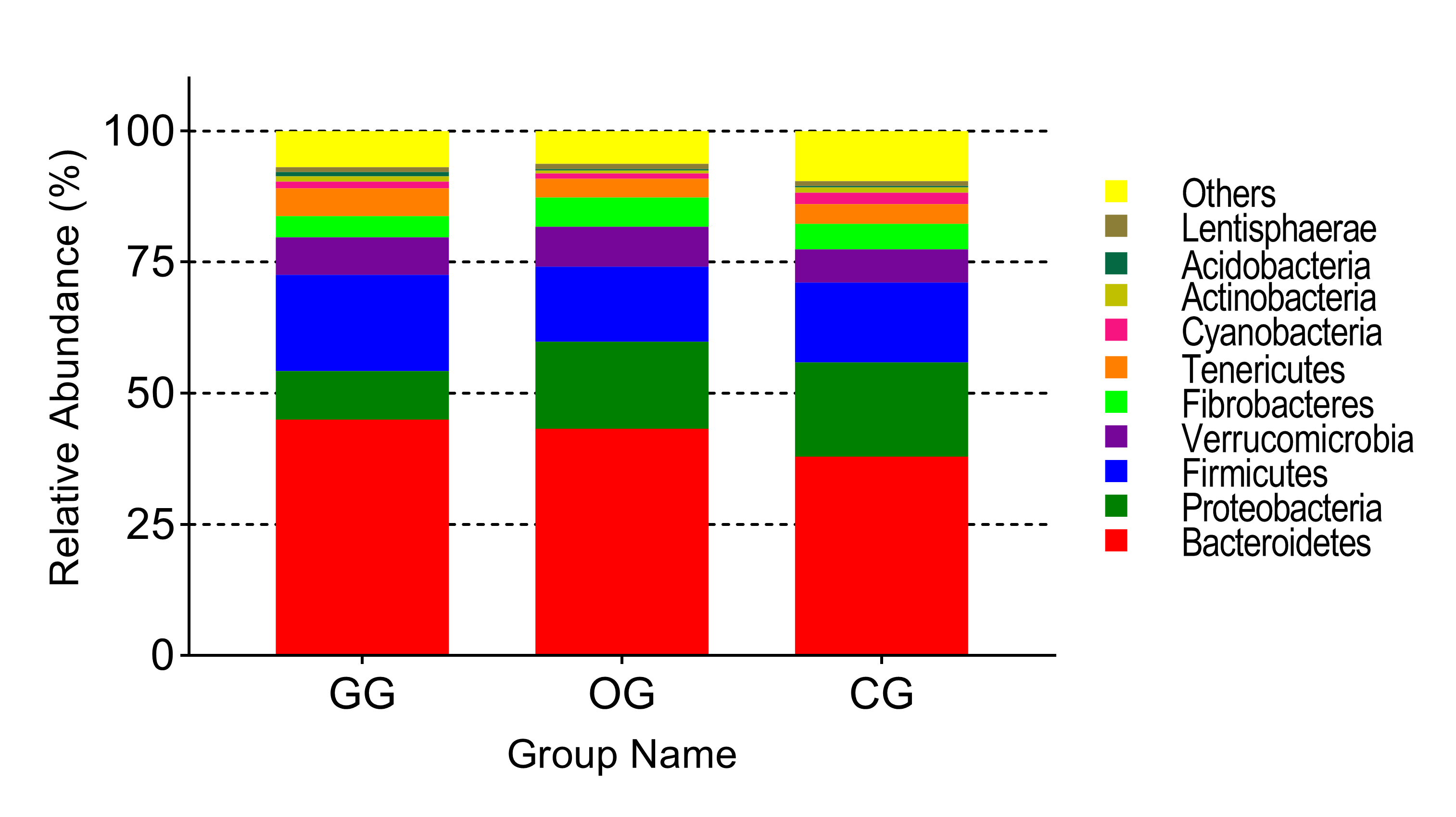

Supplement: Supplemental Information 1 — Y-axis shows the relative abundance of bacteria, while Y-axis displayed three different dietary groups. [file peerj-09-11520-s001.png]

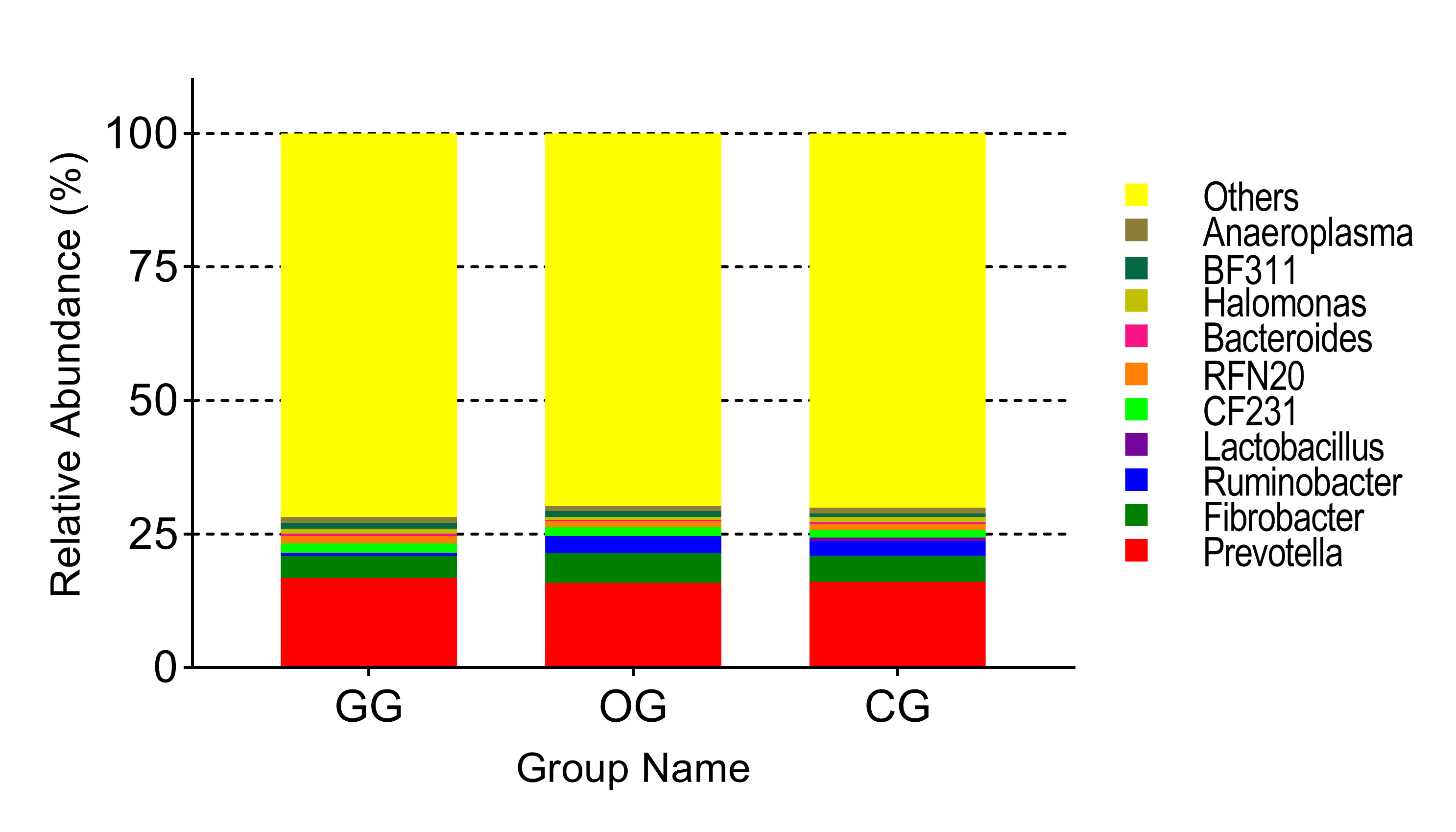

Supplement: Supplemental Information 2 — Y-axis shows the relative abundance of bacteria, while Y-axis displayed three different dietary groups. [file peerj-09-11520-s002.png]
